# Evidence for Inbreeding and Genetic Differentiation among Geographic Populations of the Saprophytic Mushroom *Trogia venenata* from Southwestern China

**DOI:** 10.1371/journal.pone.0149507

**Published:** 2016-02-18

**Authors:** Fei Mi, Ying Zhang, Dan Yang, Xiaozhao Tang, Pengfei Wang, Xiaoxia He, Yunrun Zhang, Jianyong Dong, Yang Cao, Chunli Liu, Ke-Qin Zhang, Jianping Xu

**Affiliations:** 1 Laboratory for Conservation and Utilization of Bio-Resources, and Key Laboratory for Microbial Resources of the Ministry of Education, Yunnan University, Kunming, Yunnan, PR China; 2 Institute of Communicable Disease Control and Prevention, Guizhou Provincial Centre for Disease Control and Prevention, Guiyang, Guizhou, PR China; 3 Yunnan Institute for Tropical Crop Research, Jinghong, Yunnan, PR China; 4 Kunming Edible Fungi Institute of All China Federation of Supply and Marketing Cooperatives, Kunming, Yunnan, PR China; 5 Department of Biology, McMaster University, Hamilton, Ontario, Canada; Friedrich Schiller University, GERMANY

## Abstract

During the past 40 years, more than 400 Sudden Unexplained Deaths (SUDs) have occurred in Yunnan, southwestern China. Epidemiological and toxicological analyses suggested that a newly discovered mushroom called *Trogia venenata* was the leading culprit for SUDs. At present, relatively little is known about the genetics and natural history of this mushroom. In this study, we analyzed the sequence variation at four DNA fragments among 232 fruiting bodies of *T*. *venenata* collected from seven locations. Our ITS sequence analyses confirmed that all the isolates belonged to the same species. The widespread presence of sequence heterozygosity within many strains at each of three protein-coding genes suggested that the fruiting bodies were diploid, dikaryotic or heterokaryotic. Within individual geographic populations, we found significant deviations of genotype frequencies from Hardy-Weinberg expectations, with the overall observed heterozygosity lower than that expected under random mating, consistent with prevalent inbreeding within local populations. The geographic populations were overall genetically differentiated. Interestingly, while a positive correlation was found between population genetic distance and geographic distance, there was little correlation between genetic distance and barium concentration difference for the geographic populations. Our results suggest frequent inbreeding, geographic structuring, and limited gene flow among geographic populations of *T*. *venenata* from southwestern China.

## Introduction

Since 1978, more than 400 apparently healthy persons have died suddenly without any obvious causes in Yunnan, southwestern China [1, 2]. Those Sudden Unexplained Deaths (SUDs) often occurred during the rainy season, from June to August, in remote areas surrounded by mountains at altitudes between 1800 and 2400 m [3]. This serious medical mystery attracted broad concerns from national and local governments. Various hypothetical causes have been proposed, including Keshan (a viral disease), heavy metal poisoning, drinking water contaminated with toxins or pathogens, and mushroom poisoning [1]. In China, about 3,800 species of mushrooms have been catalogued and 421 of which are considered poisonous and unfit for human consumption [4]. However, edible and poisonous mushrooms are often difficult to distinguish and incidents of mushroom poisoning are reported every year in southern China [5]. Due to the temporal clustering of SUDs around the July-August rainy season that favors mushroom fruiting and the observation that some of the households with incidents of SUDs had eaten a mushroom called the “Little White Mushroom (LWM)”, this mushroom soon became the leading culprit for SUDs. Subsequent studies identified two unusual and toxic amino acids in the fruiting bodies of LWM [6]. Indeed, recent public health campaigns warning people against consuming this and other potentially poisonous mushrooms have led to a significant reduction of SUDs in Yunnan, consistent with a potentially important role for this mushroom in SUDs. Very little, however, is known about the genetics and general biology of this mushroom.

Morphologically, the fruiting body of LWM is white when fresh and looks like a ginkgo leaf. Its fruiting body is usually about 5cm long and 3cm wide, typically found as clusters on rotten wood. Fruiting bodies of LWM are fragile and easy to break. Based on its morphological features and sequences at the internal transcribed spacer (ITS) regions of the nuclear ribosomal RNA gene, Yang et al. [7] classified this mushroom as a new species of the basidiomycete genus *Trogia* and named the LWM *Trogia venenata*. At present, this species is only found in the mountains in central and western parts of Yunnan Province.

Previous studies have shown that saprophytic fungi are generally opportunistic in their ecological distributions and are found wherever substrates (e.g. dead organic matter such as rotten wood) and growth conditions permit [8]. Indeed, frequent gene flows have been found among populations of saprophytic mushrooms (e.g. *Agaricus* species, *Lepiota cristata*, etc) [9]. Such gene flow could be accomplished through either basidiospore dispersals and/or anthropogenic activities [9]. Here, we hypothesize that populations of the saprophytic *T*. *venenata* from Yunnan will also show frequent gene flow and limited geographic differentiation.

Multilocus sequence typing (MLST) [10] and multiple gene genealogy analysis (MGGA) [11] have become popular methods for studying population biology and molecular ecology. Such studies have helped resolve a diversity of biological issues such as the modes of reproduction, cryptic speciation, gene flows, phylogeography, horizontal gene transfer, as well as ecological adaptation in a diversity of microorganisms [12, 13]. For example, genealogical analyses of DNA sequences at five mitochondrial loci and their comparisons with four nuclear loci identified evidence of horizontal gene transfer and recombination in the mitochondrial genomes of the saprophytic and opportunistic human pathogenic yeast *Cryptococcus gattii* [14]. In addition, there was abundant evidence for gene flow among geographic regions and the largely congruent phylogenetic pattern between the mitochondrial and nuclear genes suggest historical divergence and cryptic speciation within *C*. *gattii* [15, 16]. In the saprophytic mushroom *L*. *cristata*, MGGA identified long distance dispersal and recombination [9].

In this study, we collected 232 fruiting body samples of *T*. *venenata* from seven sites in Yunnan province representing the broad geographic distribution range of SUDs. ITS sequences were first obtained from representative samples at each of the seven sites to confirm species identity. These samples were then analyzed for sequence variation at the following three protein-coding genes: the second largest subunit of RNA polymerase II gene (*rpb2*), the translation elongation factor 1-α gene (*tef1-α*), and the β tubulin gene (*β-tub*). The sequences were analyzed to identify the patterns of genetic variation within and among populations. In addition, we also analyzed the putative relationships between genetic differentiation and the concentration of the heavy metal barium related to the seven sampled sites in *T*. *venenata*. A high concentration of barium in this mushroom was originally suspected as a major contributor to SUDs [1]. However, a later analysis refuted the hypothesis that this mushroom preferentially accumulated barium in its fruiting bodies and concluded that barium in *T*. *venenata* was unlikely a contributor to SUDs [17]. Despite the negative finding, much remains unknown. For example, we are still unsure if genetic distance and barium concentration difference are correlated among the geographic populations. Together, we hope these analyses will fill in a significant knowledge gap about the lead culprit of a long-standing medical mystery in southwestern China.

## Materials and Methods

### Sampling

**Fig 1:**
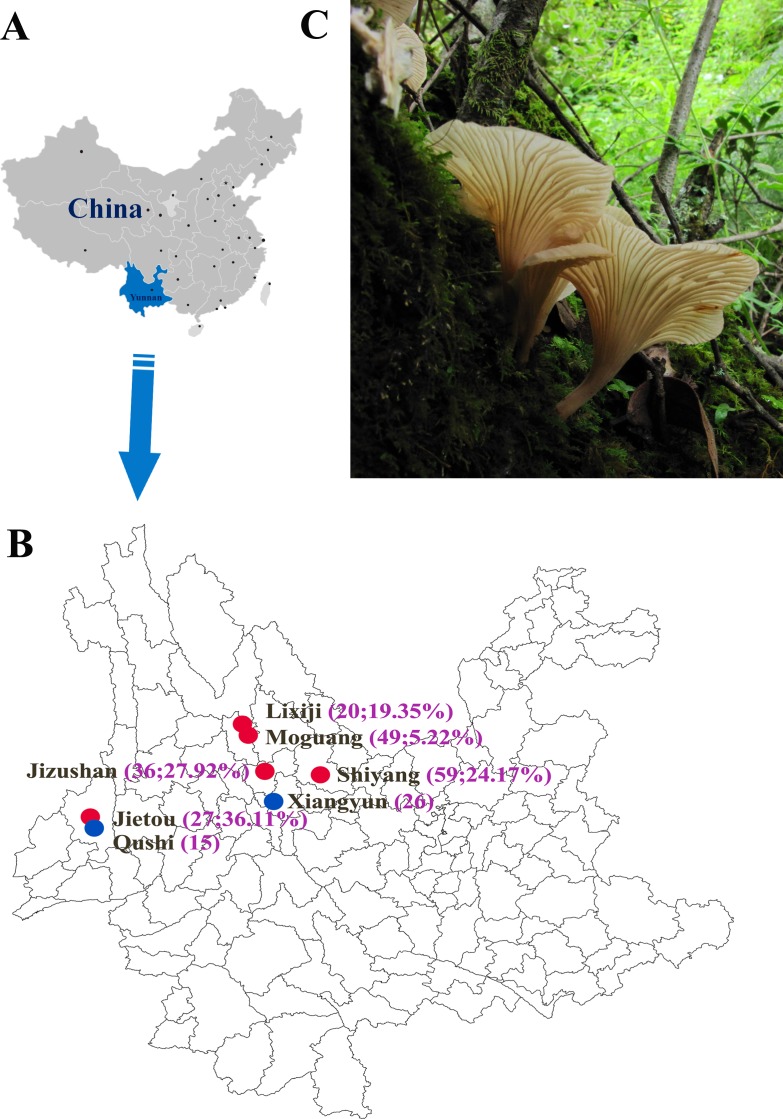
Geographic distribution of the analyzed *T*. *venenata* samples in this study. (A) Map of China and Yunnan Province is highlighted in light blue. (B) The sample collection localities (county/community) are marked in black and numbers in purple brackets represent the sample sizes and the SUD mortality rates (No. of SUDs/total population) except Xiangyun and Qushi where no SUD case was reported. Sites labeled in red had SUDs while those in blue had no SUDs. (C) A photograph of *T*. *venenata* fruiting bodies on a decaying tree stump.

Based on the patterns of SUDs from earlier epidemiological surveys, we chose seven sites for this study. These sites cover the known geographic distribution of SUDs and are located in five counties: Tengchong, Dayao, Xiangyun, Binchuan and Heqing in Yunnan Province. All seven sites are located in difficult-to-access and remote mountainous regions. The fruiting bodies of our samples were obtained from the surface of tree stumps, fallen tree trunks, and rotten wood. Of these seven sites, five were from forests close to five villages that had reported cases of SUDs while two were from communities that had never reported SUDs [18]. The seven site locations are shown in Fig 1. The collections were made possible with the help of locals who knew the fruiting sites for this mushroom. No specific permissions were required for access to the seven sampling locations. These locations were on public lands open to all people. We confirm that the field study did not involve endangered or protected species. Relevant information about each of the seven villages is presented in Table 1. A total of 232 fruiting bodies were collected during the rainy season (June-August) in 2010 and 2011. Each of the fruiting bodies was individually wrapped in clean tissue paper and dried using silica gels in sealed plastic bags. These dried mushrooms were transported from the field to the laboratory for analyses. The sample size and geographical coordinates for each population are presented in Table 1 and Fig 1.

**Table 1:** Information about the seven local populations of the mushroom *Trogia venenata* from southwestern China

					*rpb2*		*tef1-α*		*tub*		Geographic coordinate (longitude, latitude)	No. of SUDs/total population (% mortality)
Region / District	County	Village	Sample Size	Genotypic Diversity	No. of genotypes	No. of haplotypes	No. of genotypes	No. of haplotypes	No. of genotypes	No. of haplotypes		
Baoshan	Tengchong	Jietou (JT)	27	0.92	9	7	1	1	5	5	98.65°E, 25.42°N	13/36 (36.11)
	Tengchong	Qushi (QS)	15	0.93	6	6	5	4	5	7	98.6°E, 25.22°N	0/43,500 (0)
Chuxiong	Dayao	Shiyang (SY)	59	0.93	5	5	2	2	20	18	101.03°E, 25.7°N	29/120 (24.17)
Dali	Xiangyun	Xiangyun (XY)	26	0.15	2	2	1	1	2	2	100.83°E, 25.68°N	0/28,000 (0)
	Bingchuan	Jizushan (JZSH)	36	0.98	18	18	3	3	7	9	100.38°E, 25.95°N	12/43 (27.92)
	Heqing	Lixiji (LXJ)	20	0.96	8	8	4	4	3	2	100.32°E, 26.55°N	6/31 (19.35)
	Heqing	Moguang (MG)	49	0.95	5	4	3	2	10	9	100.28°E, 26.48°N	7/134 (5.22)

The term “genotype” here refers to diploid DNA sequence type at the specific locus. The term “haplotype” here refers to the number of inferred alleles using the PHASE program for the specific locus based on SNPs.

### DNA extraction and sequencing

The genomic DNA of each of the 232 samples was individually extracted from dried fruiting bodies using the CTAB method slightly modified for fungi [19]. To confirm successful extraction of DNA and assess the quantity, the genomic DNA samples were electrophoresed on a 1% agarose gel, stained with ethidium bromide and visualized under ultraviolet (UV) light. Each DNA sample was diluted to about 10ng/ml for subsequent PCR.

In this study, the following four nuclear DNA fragments were analyzed: the internal transcribed spacer (*ITS*) regions of the nuclear ribosomal RNA gene cluster, *rpb2*, *tef1-α* and *β-tub*. PCR amplifications of these four fragments were conducted using the following primer pairs: ITS4 (5' TCCTCCGCTTATTGATATGC 3') and ITS5 (5' GGAAGTAAAAGTCGTAACAAGG 3') for the *ITS* region; RPB2-577f (5' TCAGTCCAGAGGTTTCGGTTGT 3') and RPB2-577r (5' GACCGTGTCGTAGCAAGA 3') for the rpb2 gene; TEF-630f (5' CTTGTTCTAGTGGAGCGAGGAT 3') and TEF-630r (5' TTGGCAGGGTCGTTCTTC 3') for the tef1-α gene; TUB-s (5' ATCAC(A/T)CACTCICTIGGTGGTGG 3') and TUB-r (5' CATGAAGAA(A/G)TGIAGACGIGGG 3') for the β-tub gene. The ITS primers were optimized from White et al. [20], the rpb2 and tef1-α primers were optimized from the common primers used to amplify these two genes in species of the fungal phylum Basidiomycota [21], and the β-tub primers were optimized from the beta-tubulin sequences of *Psathyrella gracilis* [22]. The PCR reactions followed those described in Cao et al. [23]. PCR amplification was conducted with the following program: 4 min at 94°C, followed by 35 cycles of 30s at 94°C, 30s at 50~55°C, and 90s at 72°C, and a final extension step at 72°C for 10 min. Sequencing was performed with the dideoxy/Sanger sequencing method by BGI Co. Ltd (Shenzhen, China). DNA sequences were obtained using both the forward and reverse primers. Sequences resulting from this study are deposited in Genbank with the following accession numbers: KT967978 ~ KT968080 (*ITS*); KT971373 ~ KT971604 (*rpb2*); KT971605 ~ KT971836 (*tef1-α*); KT971837 ~ KT972068 (*β-tub*).

### Data Analysis

#### Species identification and phylogenetic analyses

Raw ITS sequences using both the forward and reverse primers were assembled for each isolate and edited using SeqMan software (DNA STAR package) [24]. These sequences were aligned using MAFFT v.6 [25] and manually checked using BioEdit v.7.0.5.2 [26]. The ITS sequence data for the isolates collected in this study were compared with those in GenBank (http://blast.ncbi.nlm.nih.gov/) using a BLAST search [27]. Here, based on the BLASTn search results (conducted in May 2015), highly similar sequences were retrieved from GenBank and aligned with our own using the MAFFT v.6 program. The aligned sequences were visually inspected, the 5' and 3' ends trimmed to ensure similar lengths, and then imported into MEGA 5.0 [28] to infer the phylogenetic relationships between our strains and those from GenBank. The Neighbor-Joining algorithm was used to construct a phylogenetic tree using the Kimura-2-parameter nucleotide substitution model [29]. Only unambiguously aligned nucleotide substitutions and insertions/deletions were included to analyze the relationships among the ITS sequences. Bootstrap support for individual branches was estimated based on 1000 repetitions.

#### Genetic diversity and population structure

Due to the multiple copy nature of *ITS* within the ribosomal rRNA gene cluster within fungi, the heterozygous sites observed within a diploid/dikaryotic individual for the ITS gene fragment could be due to variations among the copies within an individual ribosomal RNA gene cluster and not between homologous chromosomes [30]. Thus, our analyses of population structure focused on nucleotide variation at the three protein-coding genes, *rpb2*, *tef1-α* and *β-tub*. These three genes have been widely used for fungal phylogenetic and population genetic studies and they are commonly considered single-copy genes within a haploid nucleus in fungi.

At present, the mating system and reproductive life cycle of *T*. *venenata* are not known. However, in the majority of basidiomycete fungi, their sexual fruiting bodies are developed from dikaryotic or heterokaryotic mycelia. Thus, the genotype information from the nuclear genome of fruiting bodies is typically considered equivalent to a diploid. Evidence for diploidy in wild mushrooms can be found in the sequencing chromatographs. Specifically, the presence of heterozygous nucleotide sites in a sequencing chromatograph for a single-copy gene in a mushroom is commonly considered as evidence that the mushroom developed from a diploid, dikaryotic or heterokaryotic mycelia. The analyses of diploid genotypes in population genetics differ from those of haploid organisms. For example, in haploid organisms, the allele information at a single gene fragment can be directly observed from the DNA sequences. In contrast, in diploid organisms, the allele information can’t be directly observed from the polymorphic nucleotide sites due to uncertainty in the relationships among polymorphic nucleotide sites within each sequenced gene fragment.

Thus, to analyze the three nuclear gene sequence information fully, we first inferred the putative haplotype sequences for each individual mushroom at each of the three nuclear loci respectively using the Bayesian method implemented in the program PHASE 2.1 [31]. The inferred haplotype sequences were then used to analyze the phylogenetic relationships among alleles from within the same and different mushroom fruiting bodies using the maximum parsimony algorithm implemented in PAUP*4.0b10 [32]. In addition, the haplotype information was combined into one dataset and imported into GenAlEx version 6.5 [33] to calculate the pairwise population *F*_*ST*_ values [34] and determine the potential correlation between genetic and geographical distances. The total genetic variation is partitioned at three levels: within local populations (Phi-PT), among local populations within regions (PhiPR), and among regional populations (Phi-RT) [35]. The local populations were defined as the seven sampling sites while the three regional populations were classified based on their political/administrative boundaries (a regional level of government is between the county and the provincial governments): Baoshan, Dali, and Chuxiong (Table 1). The analysis of molecular variance (AMOVA) was performed to estimate the relative contributions of geographic and political/administrative separations to the overall genetic variation [36].

Aside from the above population genetic analyses, we also estimated the number of genetic clusters using the program STRUCTURE version 2.3.3 [37]. The STRUCTURE program uses a Markov Chain Monte Carlo (MCMC) algorithm to cluster individuals into genetic populations on the basis of multilocus genetic data [38, 39], and it has been applied to solve problems such as identifying cryptic population structure, detecting migrants and inferring historical population admixture [40, 41]. Generally, a broad range of clusters (K) are tested and Hardy-Weinberg equilibrium (HWE) and linkage equilibrium are assumed within individual genetic clusters, the program STRUCTURE then estimates allele and genotype frequencies in each cluster and provide the likely population memberships for every individual. Individuals were assigned to clusters to maximize HWE and linkage equilibrium within each cluster. The true number of populations (K) is commonly identified using the maximal value of L(K) returned by STRUCTURE [42]. However, the real K may not be identified during simulations if the distribution of L (K) did not show a clear mode for the true K. Instead, Delta K(∆K), a statistic based on the rate of change in the log probability of data with respect to the number of clusters, is typically used to derive the number of genetic clusters [43]. In our analyses, a total of 10 simulations were performed for K ranging from 1 to 10 to verify the convergence of the Log likelihood values for each value of K. After the optimal K was determined, a final parameter of 1 million MCMC replicates and a length of burn-in period of 100,000 were run for the assignment of individuals into K populations. Here, we used CLUMPAK (Cluster Markov Packager Across K) to process the STRUCTURE output files to obtain the optimal number of K in our samples, following the Evanno method [43] [44]. CLUMPAK is available at http://clumpak.tau.ac.il and our STRUCTURE output files were uploaded as a compressed file (.zip) to the site directly to conduct the analysis.

#### Tests of linkage equilibrium and Hardy-Weinberg equilibrium

The modes of reproduction for fungi in nature are commonly inferred based on the allelic relationships in natural populations. In diploid/dikaryotic/heterokaryotic organisms, there are two types of allelic relationships. The first is between alleles from the same locus, the commonly known Hardy-Weinberg equilibrium. The null hypothesis of the HWE test is that the observed genotype frequencies at each locus are not significantly different from those expected under the null model of random association. Here in this study, tests of HWE at each locus for each geographic population were conducted using the program GenAlEx version 6.5. For loci that showed significant deviations from HWE, we further compared whether the observed heterozygosity was significantly higher or lower than those expected under random mating. In this test, Ho is the observed heterozygosity, He is the expected heterozygosity and the Fixation Index F (also called the Inbreeding Coefficient) exhibits values ranging from -1 to +1. Values close to zero are as expected under random mating, while substantial positive values indicate inbreeding. Negative values indicate excess of heterozygosity, due to negative assortative mating or significant heterozygote advantage.

The second test is linkage equilibrium, assessing the associations among alleles at different loci. In diploid organisms, the test is also called genotypic equilibrium where the observed allelic relationships within a locus are fixed and only associations among the diploid genotypes at different loci are tested. Fixing the diploid genotype at each locus in this test is to eliminate the effects of potential deviations of HWE on the genotypic equilibrium calculations. The program MULTILOCUS version 1.3b [45] was used to estimate genotypic equilibrium in local natural populations of *T*. *venenata*. In our tests, because the geographic samples are genetically differentiated (see below in Results), each local geographic sample was tested separately to avoid the Wahlund effect. Two tests were conducted for each geographic population using both the total sample and the clone-corrected sample: the phylogenetic compatibility and the multilocus index of association (I_A_). Because I_A_ can be influenced by the number of polymorphic loci (typically the higher the number of loci, the higher the I_A_ value), we also standardized the I_A_ value by the number of loci to generate the rBarD value for comparisons among populations. The null hypothesis for I_A_ is that there is random association (recombination, linkage equilibrium) among alleles (or diploid genotypes in our case) at different loci, a P-value of <0.05 would indicate that the null hypothesis should be rejected. Phylogenetic incompatibility is another indicator of recombination at the population level. The observed value was compared to that of 500 randomizations in which individuals were randomized across populations.

#### Testing the relationship between genetic differentiation and barium concentration difference among the geographic populations

DNA sequence divergence among populations was estimated using pairwise *F*_*ST*_ values. The quantitative relationship between genetic difference and geographic distance were assessed using the Mantel test [46]. The geographic distances between local populations were calculated between each pair of populations based on their geographic coordinates at each location. The relationships between population genetic distances and geographic distance were assessed using the GenAlEx version 6.5 program. In addition, we also analyzed the relationships between genetic differentiation and differences in barium concentration among the sites using the Mantel test implemented in GenAlEx.

#### GenBank accession numbers

The GenBank accession numbers for the sequences generated in this study are KT967978 to KT968080 for the internal transcribed spacer (ITS) regions of the nuclear ribosomal RNA gene cluster; KT971373 to KT971604 for the second largest subunit of the nuclear RNA polymerase B gene (rpb2); KT971605 to KT971836 for the translation elongation factor 1-α gene (tef1-α); and KT971837 to KT972068 for the β tubulin gene (β-tub).

## Results

### Sequence analysis and ITS identification

**Fig 2:**
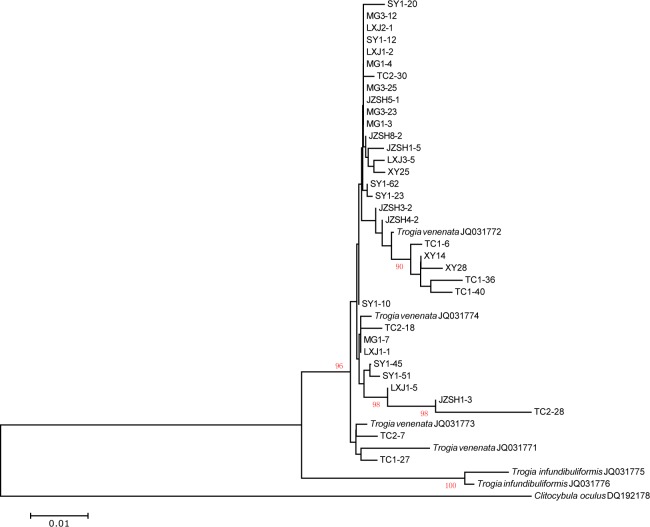
A Neighbor-Joining tree showing the relationships among 35 representative ITS sequences from seven local populations of *T*. *venenata* from Yunnan. The *T*. *venenata* type specimen sequences as well as those of a closely related species retrieved from GenBank were included. The reference strains are each represented by their species name and the GenBank accession number. The bootstrap values were obtained from 1000 replicates. Only bootstrap values greater than 90% are shown. *Clitocybula oculus* is used as the outgroup.

Among the 232 fruiting body specimens that we sequenced for ITS, we were able to get clean chromatograms from 103 isolates. Of the 129 isolates that had unreadable ITS sequences, we further cloned the PCR products from 15 isolates and then sequenced one cloned ITS fragment from each isolate. Alignment of the 118 ITS sequences identified 38 variable nucleotide sites, including 24 indels and 14 single nucleotide base substitutions. These sequences were compared with those in GenBank. Our BLAST comparisons showed that our ITS sequences were most similar to those belonging to *T*. *venenata* (GenBank accession numbers: JQ031772, JQ031773 and JQ031774). Fig 2 shows a Neighbour-Joining tree of our representative ITS sequences with those from the type specimens of *T*. *venenata* and its closely related species *Trogia infundibuliformis*. Here, we used the ITS sequence of *Clitocybula oculus* as the outgroup. Only bootstrap values greater than 90 are shown for the NJ tree. Our results showed that one ITS sequence type was found in five geographic populations (MG, LXJ, SY, and JZSH) while most others were geographically more limited (Fig 2).

### Genetic variation within the sequenced single-copy gene fragments

In this study, we successfully obtained sequences from all three protein-coding genes analyzed here for the 232 specimens. The polymorphisms found in each of the three gene fragments are briefly described below. Three corresponding maximum parsimony (MP) trees containing all the representative haplotype sequences for the three gene fragments in each population, with one tree for each of the three gene fragments, were obtained using PAUP*4.0b10 (S1–S3 Figs). The Kimura-2-parameter nucleotide substitution model was used. Since the sequences were all from within the same species, the nucleotide differences among the haplotypes were minor and there were very low bootstrap values for all the branches.

Among the 497 aligned nucleotide sites for the *rpb2* gene fragment, we found 19 variable sites. PHASE analyses identified a total of 45 haplotypes among these 232 strains. None of the 45 haplotypes at the *rpb2* locus were shared by all seven geographic populations. The most frequent haplotype was HAP24 and it was found in four local populations (JZSH, MG, JT and QS). The second most frequent haplotype was HAP30 and most of the strains in the XY population shared this haplotype. Six of the 45 inferred haplotypes were found only once each. The phylogenetic relationships among the haplotypes are presented in S1 Fig.

For the *tef1-α* gene fragment, the 563 aligned nucleotides contained seven variable sites and a total of eight haplotypes in the *T*. *venenata* populations. Among them, HAP2 was the most frequent, representing about 70% (324/464) of all the haplotypes at this locus and it was distributed in six of the seven local populations except for JT. However, more than one third of HAP2 was found in the SY population. Another frequent haplotype was HAP4, most abundantly distributed in the JT population but also found in four other local populations. Five of the eight haplotypes (i.e. HAP3, HAP5, HAP6, HAP7, and HAP8) were only found in one local population each, with HAP3 found only in one fruiting body in the LXJ population (S2 Fig).

For the *β-tub* gene fragment, 440 nucleotide sites were aligned and analyzed. A total of 18 variable sites were found and PHASE analyses inferred a total of 41 haplotypes. The most frequent haplotype was HAP1 and it was found in four populations, followed by HAP4. The SY population had the highest number of haplotypes, with a total of 20 haplotypes. Seven of the 41 haplotypes were found only once each. The relationships among the haplotypes and their geographic distributions are shown in (S3 Fig).

### Genetic differentiation and structure of the geographical populations

The haplotype information obtained above was used to analyze the population structure of *T*. *venenata* from Yunnan province. To identify the overall population structure, we analyzed the combined dataset of the three sequenced genes (*rpb2*, *tef1-α* and *β-tub*).

Our analyses identified abundant genetic variations within each of the seven local populations. The overall genotypic diversity for the whole sample of 232 individuals was 0.83, suggesting that over 83% of the time, two randomly drawn fruiting bodies from the total population will have genotypes different in at least one of the assayed loci. However, a range of genotypic diversity was found among the seven local populations, from a low of 0.15 in Xiangyun to a high of 0.98 in Jizushan (Table 1). The results from these analyses suggested that the seven local populations differed in their genetic diversities and that there are high levels of genetic variations at the three loci within most of the local populations.

**Fig 3:**
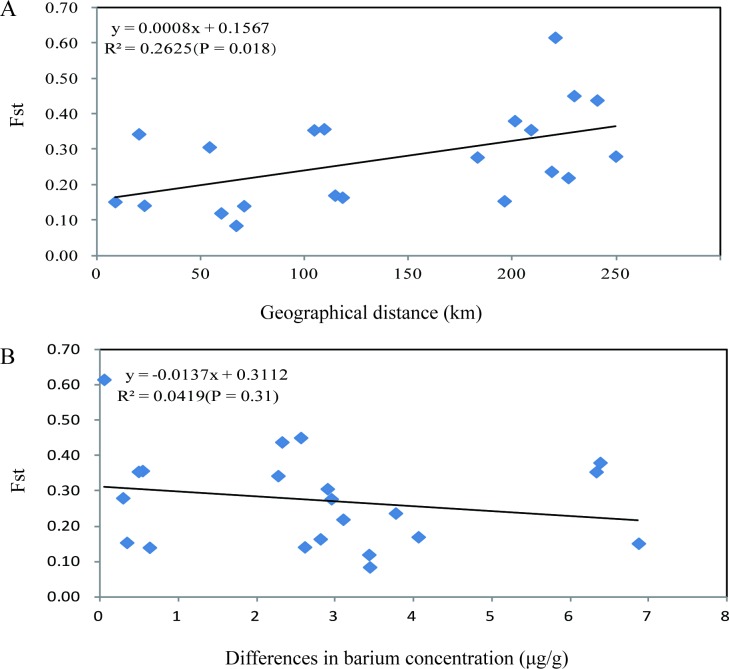
Mantel tests of the relationships among genetic differentiation (*F*_*ST*_ values), geographical distance, and differences in barium concentration among the seven local populations of *T*. *venenata* from Yunnan, southwestern China. (A) Mantel test between *F*_*ST*_ values and the geographical distance among populations (P = 0.018). (B) Mantel test between *F*_*ST*_ values and barium concentration differences among populations (P = 0.31).

Similarly, a range of *F*_*ST*_ values between pairs of local populations was found (S1 Table). The *F*_*ST*_ values between populations of *T*. *venenata* varied from 0.084 to 0.615. The lowest value (0.084) was found between Jizushan and Lixiji, separated by about 67 kilometers, while the highest (0.615) was found between Xiangyun and Jietou, separated by about 221 kilometers. Overall, the Mantel test results showed a significant positive correlation between genetic difference and geographical distance among these seven populations (P = 0.018) (Fig 3A).

The results of AMOVA also suggested significant genetic variations within populations (S2 Table). The analysis based on the dataset of three genes revealed that 51% of the genetic variation was found within individual populations (PhiPT). However, 36% of the total genetic variance (PhiPR) could be attributed to among local populations within regions and the remaining 14% of the total genetic variance (PhiRT) was attributed to among regional populations. All of the three levels contributed significantly to the overall genetic variation, as determined through the permutation analyses.

**Fig 4:**
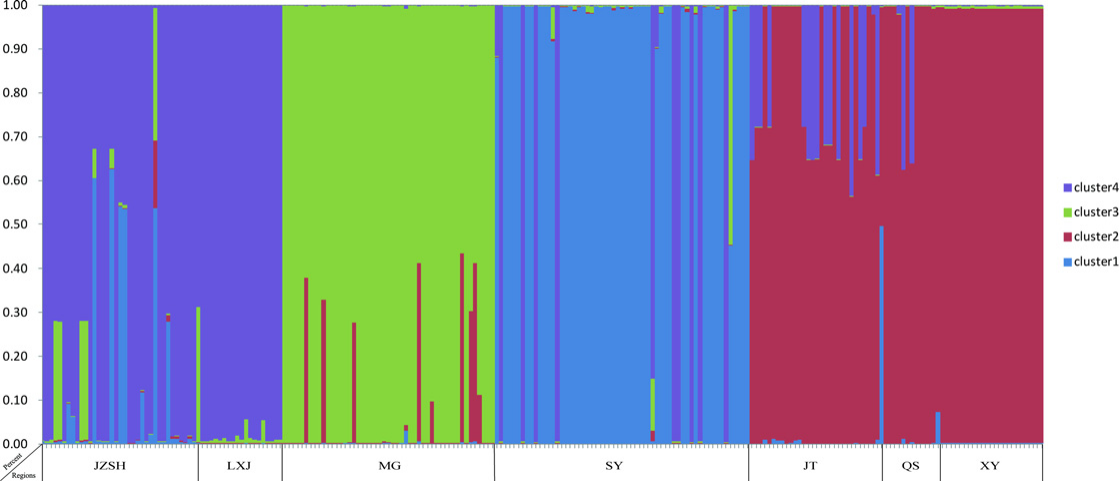
Genetic clusters and individuals’ ancestry as inferred from Bayesian clustering with STRUCTURE based on SNPs. The x-axis represents geographic locations where the isolates were collected. The y-axis represents the assignment probability of each isolate to one of the four genetic clusters that are colored differently. The optimal number of genetic clusters identified here was 4. Each individual isolate is represented by one line and populations were marked on the bottom. Details of the analyses are presented in the main text.

The number of clusters K = 4 was inferred because the standard deviation of posterior probability was the lowest for that K (S4 Fig). Our analysis using data from the 44 SNPs revealed evidence of strain clustering based on their geographic origins (Fig 4). Specifically, two local populations, Jizushan (JZSH) and Lixiji (LXJ), separated by 67 km were clustered together, while three other populations [from Jietou (JT), Qushi (QS) and Xiangyun (XY), separated by an average geographic distance of 220 kilometers] formed one cluster. The remaining two local populations (MG and SY) were genetically distinct from all others based on STRUCTURE analyses.

### Mode of reproduction

Based on the genotypes and allele frequencies for the seven populations, we assessed whether the local populations were in HWE, as would be expected if the local populations were randomly mating. Our overall results rejected the null hypothesis of HWE for each of the seven local populations (S3 Table). We then further examined the observed heterozygosity, expected heterozygosity and the Fixation Index at each locus for each population. As showed in Table 2, all seven local populations had positive F values at the *rpb2* locus, with the observed heterozygosities lower than those expected under the hypothesis of random mating. For the *tef1-α* locus, two populations (JT and XY) were monomorphic, one population (SY) showed slight excess of observed heterozygosity while four (JZSH, LXJ, MG, and QS) had excess of homozygotes. For the *β-tub* locus, four local populations (MG, SY, JT and XY) showed excess homozygosity while three (QS, JZSH and LXJ) had slight excess of heterozygosity.

**Table 2:** Evidence for inbreeding in populations of *T*. *venenata* from southwestern China.

Population	Locus	Observed Heterozygosity (Ho)	Expected Heterozygosity (He)	Fixation Index (F value)
Jizushan	*rpb2*	0.278	0.889	0.688
	*tef1-α*	0.111	0.512	0.783
	*β-tub*	1.000	0.837	-0.194
Lixiji	*rpb2*	0.050	0.786	0.936
	*tef1-α*	0.150	0.404	0.628
	*β-tub*	0.700	0.480	-0.458
Moguang	*rpb2*	0.061	0.461	0.867
	*tef1-α*	0.224	0.259	0.134
	*β-tub*	0.735	0.780	0.058
Shiyang	*rpb2*	0.017	0.341	0.950
	*tef1-α*	0.102	0.097	-0.054
	*β-tub*	0.559	0.897	0.377
Jietou	*rpb2*	0.222	0.841	0.736
	*tef1-α*	0.000	0.000	#N/A
	*β-tub*	0.185	0.567	0.674
Qushi	*rpb2*	0.000	0.604	1.000
	*tef1-α*	0.467	0.658	0.291
	*β-tub*	0.800	0.796	-0.006
Xiangyun	*rpb2*	0.000	0.074	1.000
	*tef1-α*	0.000	0.000	#N/A
	*β-tub*	0.000	0.074	1.000
Mean		0.270	0.493	0.495

In addition to HWE tests, the SNPs were also analyzed for their associations with each other using the MULTILOCUS software. SNPs located within the same DNA fragment were considered genetically linked. In this analysis, each variable nucleotide site was treated as a locus and different nucleotides at the same site were viewed as different alleles. The results of our analyses were consistent with recombination in natural populations of *T*. *venenata*. For the total samples, the observed rBarD value was 0.049 (P = 0.962), and for the clone-corrected sample, it was 0.046 (P = 0.974) (Table 3). These results suggest evidence for recombination among the gene loci in the total population in nature. Similar results were found at most local populations (Table 3). However, evidence for genotypic disequilibrium was also found in some of the populations (SY, JT, and QS; Table 3), consistent with a certain degree of local clonality. Taken together, our results suggest that populations of *T*. *venenata* from southwest China are mating and recombining, but with inbreeding (lower observed heterozygosity than expected) as an important component of their reproduction that generates significant HW disequilibrium within loci and linkage disequilibrium among loci.

**Table 3:** Results of multilocus linkage disequilibrium analyses for populations of *T*. *venenata*.

Population	Phylogenetic compatibility (P value)	I_A_ (P value)	rBarD (P value)
Total sample (n = 232)	0.646 (< 0.002)	1.735 (0.962)	0.049 (0.962)
Total clone-corrected (n = 126)	0.646 (< 0.002)	1.663 (0.974)	0.046 (0.974)
JZSH (n = 36)	0.841 (0.094)	2.570 (0.040)	0.106 (0.040)
JZSH clone-corrected (n = 27)	0.841 (0.082)	2.103 (0.252)	0.087 (0.252)
LXJ (n = 20)	0.988 (0.138)	0.944 (0.422)	0.099 (0.422)
LXJ clone-corrected (n = 16)	0.988 (0.090)	0.957 (0.524)	0.098 (0.524)
MG (n = 49)	0.983 (0.278)	0.767 (0.502)	0.095 (0.502)
MG clone-corrected (n = 30)	0.983 (0.114)	0.521 (0.886)	0.064 (0.886)
SY (n = 59)	0.956 (< 0.002)	6.907 (< 0.002)	0.395 (< 0.002)
SY clone-corrected (n = 28)	0.956 (< 0.002)	5.226 (< 0.002)	0.289 (< 0.002)
JT (n = 27)	0.995 (< 0.002)	2.643 (< 0.002)	0.196 (< 0.002)
JT clone-corrected (n = 12)	0.995 (< 0.002)	2.220 (0.034)	0.161 (0.034)
QS (n = 15)	0.960 (< 0.002)	4.780 (0.016)	0.237 (0.016)
QS clone-corrected (n = 10)	0.960 (< 0.002)	3.509 (0.006)	0.173 (0.006)
XY (n = 26)	1.000 (1.000)	-0.040 (1.000)	-0.040 (1.000)
XY clone-corrected (n = 3)			

I_A_, the index of association, a multilocus measure of linkage disequilibrium (If there are no associations between loci, then all these covariances are expected to be not significantly different from zero. rBarD, a standardized measure of multilocus linkage disequilibrium (I_A_/n, where n equals the number of analyzed loci).

### Characteristics of barium concentration differences associated with *Trogia venenata*

The potential relationship between genetic distance and barium concentration difference among the *T*. *venenata* populations from Yunnan province is unknown. Here, we evaluated the relationships between these two variables to examine whether there is any potential correlation between them for the seven local populations. Our Mantel test showed a slightly negative correlation between levels of barium concentration and their genetic distances (Fig 3B). However, the correlation was statistically insignificant (P = 0.31).

## Discussion

In this study, we obtained DNA sequences from 232 specimens at each of the three protein-coding genes. The sequence information was used to analyze the diversity and population structure within and among geographic samples of *T*. *venenata* in southwestern China. There was abundant sequence variation within and among isolates at the three protein-coding genes and the geographic populations were overall genetically differentiated. While a positive correlation was found between population genetic distance and geographic distance, there was little correlation between genetic distance and barium concentration for the geographic populations. Below we discuss the relevance of our results to general biology of this and other mushrooms.

### ITS sequence variation

All four gene fragments (*ITS*, *rpb2*, *tef1-α* and *β-tub*) that we sequenced were polymorphic in our samples. Among these four fragments, the *ITS* fragment from direct PCR and sequencing showed a large number of heterozygous sites in chromatograms in 129 of the 232 isolates (55.6%), rendering their chromatograms unreadable for significant portions of the gene fragment. The unreadable chromatographs were not due to base substitutions at one or a few single nucleotide sites, because base substitution polymorphisms are almost universally readable as was shown in our sequences of the three protein-coding genes. Rather, they were due to prevalent insertions and deletions, as shown by the alignments of the 118 *ITS* sequences (103 from direct PCR sequencing and 15 from cloning the PCR product first followed by sequencing). The high failure rate (55%) in direct sequencing of PCR products of the *ITS* suggested that this locus is not a good barcode for species identification of *T*. *venenata* using PCR amplification followed by traditional Sanger sequencing method. However, the frequent within-strain *ITS* sequence heterogeneity should not be a problem for barcode identification if next-generation sequencing methods such as Roche 454 and Illumina are used. Given that the dominant approach for *ITS* sequence-based specimen identification of fungi is Sanger sequencing, other markers e.g. the three protein-coding genes analyzed here, should be explored as alternative barcode markers. Specifically, the patterns of intra- and inter- species sequence variation should be analyzed at these loci for the taxa closely related to *T*. *venenata* to help define the barcode gap among the closely related species in this genus.

The *ITS* regions are located within the multi-copy nuclear ribosomal RNA gene cluster. This gene cluster is known to undergo concerted evolution and that different copies of the rRNA subunits are typically identical to each other within an individual organism or even a species. Thus, the high rate of sequence heterozygosity at the *ITS* for many fruiting bodies of *T*. *venenata* in our sample was unexpected. At present, the detailed mechanisms for such a high *ITS* sequence heterogeneity is not known. Given the diploid, dikaryotic, or heterokaryotic nature of *T*. *venenata* fruiting bodies, there are two potential mechanisms for the high *ITS* sequence heterogeneity. The first is that the observed *ITS* sequence heterozygosity could be due to *ITS* sequence differences among copies within an rRNA gene cluster. The second reason is that the two different clusters located on the two homologous chromosomes within the 129 fruiting bodies were different from each other. Given the low observed heterozygosities at the three protein coding genes, the first mechanism is likely playing a more important role than the second for the high rate of *ITS* sequence heterogeneity within individual strains. Our results thus suggest that concerted evolution doesn’t seem to be very effective at homogenizing the *ITS* sequences within individuals of *T*. *venenata*.

### Sequence variation at other loci

We found a high number of haplotypes for each of the three protein-coding DNA fragments. For each gene fragment, we found abundant homozygotes, consistent with these three loci as single copy genes in *T*. *venenata*. Among the three loci, *rpb2* and *β-tub* were more polymorphic than *tef1-α* gene, consistent with results from an earlier study by Matheny [21] that showed the *rpb2* gene as being more variable than *tef1-α* in Basidiomycota. Among the seven local populations, the lowest genotypic diversity was found in the XY population (0.15) while the remaining six geographical populations each had genotypic diversities ranging from 0.92 for the JT population in Tengchong to 0.98 for the JZSH population in Binchuan (Table 1). For each of the three loci, we identified several haplotypes that were found in only one of the local populations. These results indicated abundant haplotype variations with both shared and unique haplotypes at the seven local populations.

### Genetic differentiation and population structure among geographic populations

Our analyses identified significant genetic differentiation among the seven local populations analyzed in this study. The observed *F*_*ST*_ values between pair of populations ranged from 0.084 to 0.615 and the AMOVA results showed that 36% of the total genetic variance was attributed to among local populations within regions and 14% of the total genetic variance attributed to among regional populations. Interestingly, the genetic differentiations observed here for *T*. *venenata* were higher than most other mushrooms from southwestern China. For example, in the ectomycorrhizal basidiomycete *Thelephora ganbajun*, the genetic variations contributed by differences among local and region populations were 7.4% and 10.1% respectively from diverse regions in Yunnan [47]. For the gourmet ectomycorrhizal mushroom *Tricholoma matsutake*, the proportions of genetic variation attributed to differences among local and regional populations were 34% and 9% respectively [35]. Furthermore, our observed *F*_*ST*_ values were higher than those found among regional populations in the saprophytic button mushroom *Agaricus bisporus* [48] and in *Pleurotus eryngii* var. *tuoliensis* [49]. The local and regional populations of *A*. *bisporus* were separated by greater geographic distances than our *T*. *venenata* samples [48]. Indeed, the results contrasted our original expectation that the saprophytic mushroom *T*. *venenata* should show limited geographic differentiations among local and regional populations.

Similar to results from these studies [47–51], our analyses showed a significant positive correlation between genetic difference and geographical distance. Positive correlation between genetic distance and geographic distance has also been found in other saprophytic fungal species, including species in the *Pleurotus eryngii* complex [52]. Thus, our results here and the results from previous studies indicate that geographical isolation is an important factor for the genetic differentiation in *T*. *venenata* and many other fungal species in nature.

Our STRUCTURE results were overall consistent with the *F*_*ST*_ and AMOVA results. However, there was one notable difference. Specifically, the STRUCTURE results suggested that all our samples could be grouped into four genetic clusters, not seven local clusters based on their geographic origins. This incongruence was likely caused by differences between the two types of analyses. The genetic differentiation analyses (*F*_*ST*_ and AMOVA) were mainly based on haplotype and genotype frequencies at the analyzed loci. However, STRUCTURE analyses were mainly based on the patterns of allelic and haplotype associations within and among individuals. The different results in the two approaches can be shown by the relationships between two local populations, Jietou and Qushi, from Tengchong. These two populations were located very close to each other. *F*_*ST*_ analysis showed that they were statistically significantly differentiated due to differences in allele frequencies. However, STRUCTURE analyses grouped them into the same genetic cluster due to their shared alleles and haplotype associations at the three analyzed loci.

### Gene flow and geographic barriers

Despite the observed genetic differentiation, our analyses based on data from the three gene fragments (haplotype inferences and AMOVA test) also showed evidence for gene flow. Specifically, the haplotype comparisons identified that each of the three loci (*rpb2*, *tef1-α* and *β-tub*) had haplotypes that are broadly distributed among several local populations. For example, at the *rpb2* locus, HAP24 was shared among four local populations (JZSH, MG, JT and QS) while HAP30 was found in three local populations. At *tef1-α*, HAP2 was found in six of the seven local populations while HAP4 was distributed among five geographic populations. Similarly, HAP1 of the *β-tub* locus was distributed in four local populations (JZSH, LXJ, MG and SY).

Southwestern China is known for its big mountains, deep gorges, and fast rivers. Such geographic barriers are known to impact the dispersal of land animals [53]. However, the effects of such barriers on gene flow in mushroom fungi have remained controversial. Our results here and those from earlier studies on *L*. *cristata* [9], *R*. *virescens* [23], *Thelephora ganbajun* [47], ‘‘Big Red Mushroom” [50] and *T*. *matsutake* [35] suggest that species and populations of mushrooms differ in their dispersal abilities. For example, in *T*. *venenata*, while haplotype sharing was common among the geographic populations, their pairwise *Fst* values were all statistically significant, consistent with some barriers to gene flow among the populations. Unlike the edible ectomycorrhizal species such as *R*. *virescens* [23], *Thelephora ganbajun* [47], ‘‘Big Red Mushroom” [50], and *T*. *matsutake* [35] where anthropogenic activities could have facilitated spore and mycelia dispersals from mushroom trading, *T*. *venenata* is a poisonous mushroom not found in mushroom markets. Thus, *T*. *venenata* is unlikely to have been dispersed by anthropogenic activities, possibly contributing to their greater genetic differentiations.

### Inbreeding and recombination for the geographic populations

At present, there is no information about the mating system and reproductive behavior of *T*. *venenata*. The discovery of both homozygous and heterozygous genotypes at all three protein-coding gene loci suggested that sexual reproduction, including both mating and segregation, is likely common in natural populations of *T*. *venenata*. However, the lower observed than expected heterozygosities at most loci in most local populations indicated two possibilities. In the first, mating in *T*. *venenata* is primarily between monokaryotic or homokaryotic mycelia that are genetically closely related to each other. The genetic effect of such inbreeding would result in higher than expected homozygosities. The second possibility is that mating is random in nature but there is a high selective pressure against heterozygotes. Giving that four of the locus-population combinations had excess heterozygote or that their genotype frequencies were not significantly different from those expected under random mating, selective pressure against heterozygotes, if exists, is unlikely to be prevalent to cause such broad heterozygote deficiencies we observed here. Instead, we believe the first hypothesis (i.e. inbreeding) is the more likely explanation. In addition, the excess heterozygotes observed at the few locus-population combinations were likely due to selection against homozygotes. For example, in the saprophytic button mushroom *Agaricus bisporus*, homozygote disadvantage has been demonstrated, consistent with inbreeding depression [54].

### SUDs associated with *T*. *venenata*

We observed no association between genetic distance and barium concentration difference. Our result further suggested that barium is unlikely an important factor in the biology and toxicity of these mushrooms. The current evidence suggests that two unusual amino acids, 2*R*-amino-4*S*-hydroxy-5-hexynoic acid and 2*R*-amino-5-hexynoic acid, are likely the main contributors to the toxic effects of *T*. *venenata* in humans [6, 55]. However, as discussed in Zhang et al. [17], the two amino acids in *T*. *venenata* could not explain all the SUDs cases. Other factors such as human host genotypes and environmental factors were also likely involved. Even with the two toxins, we know almost nothing about the genes and molecular processes involved in their synthesis and their detailed toxicity mechanisms.

Previous studies have shown that genetically divergent populations of human fungal pathogens such as *Candida albicans* [56], *Cryptococcus gattii* [15, 57], and *Aspergillus terreus* [58] can differ in their pathogenicity and virulence in humans and animals. At the population level, we don’t know whether different populations of *T*. *venenata* contain different concentrations of these two toxic amino acids. And, if the toxin concentrations were different, could they explain the differences in SUD mortality rates among the communities? Because these two amino acids are presented at very low concentrations in fruiting bodies of *T*. *venenata* [6], large amounts of fruiting bodies (several kilograms) are needed in order to extract sufficient amounts for analyses and quantification. However, due to the small size of individual fruiting bodies, we were unable to obtain accurate concentration information of the two amino acids for individual fruiting bodies analyzed here.

## Conclusions

In conclusion, the newly discovered poisonous mushroom *T*. *venenata* from southwest China is a diploid, dikaryotic or heterokaryotic species. Among the four sequenced gene fragments, the *ITS* fragment had a large number insertions and deletions, making the *ITS* sequence chromatographs from direct PCR and sequencing reactions of many fruiting bodies unreadable. There was abundant sequence variation within and among isolates at the three protein-coding genes and the geographic populations were overall genetically differentiated. Our analyses suggest prevalent inbreeding and low levels of gene flow among the local populations. The lack of statistically significant correlation between population genetic distance and barium concentration difference among the seven geographic populations of *T*. *venenata* suggest that the genetic differentiation observed at the three loci among local populations is unlikely a significant contributor to the toxic effects of *T*. *venenata* in humans.

## Supporting Information

S1 Fig A maximum parsimony tree of the representative *rpb2* gene sequences of the *T*. *venenata* species in Yunnan, southwestern China. A total of 45 unique rpb2 haplotypes were found in our samples. For each rpb2 haplotype (HAP), the first number represents the haplotype assignment; the characters after represent the county/community from where the strains were sampled; the last number represents the total number of strains belonging to the specific haplotype in that local population. Only representative sequences of unique haplotypes from each location are shown.
(TIF)

S2 Fig A maximum parsimony tree of the representative *tef1-α* gene sequences of the *T*. *venenata* species in Yunnan, southwestern China. A total of 8 unique tef1-α haplotypes were found in our samples. For each tef1-α haplotype (HAP), the first number represents the haplotype assignment; the characters after represent the county/community from where the strains were sampled; the last number represents the total number of strains belonging to the specific haplotype in that local population. Only representative sequences of unique haplotypes from each location are shown.
(TIF)

S3 Fig A maximum parsimony tree of the representative β-tub gene sequences of the *T*. *venenata* species in Yunnan, southwestern China. A total of 41 unique β-tub haplotypes were found in our samples. For each β-tub haplotype (HAP), the first number represents the haplotype assignment; the characters after represent the county/community from where the strains were sampled; the last number represents the total number of strains belonging to the specific haplotype in that local population. Only representative sequences of unique haplotypes from each location are shown.
(TIF)

S4 Fig Plots for detecting the number of K genetic groups that best fit the data. (TIF)

S1 Table Pairwise *F*_*ST*_ values between geographical populations of *T*. *venenata* from Yunnan, southwestern China. (DOCX)

S2 Table Summary results of AMOVA within and among geographic populations of *Trogia venenata* from Yunnan, southwestern China. (DOCX)

S3 Table Hardy-Weinberg Equilibrium tests for each geographic population at each locus. (DOCX)

S4 Table GenBank accession numbers for sequences presented in this study. (DOCX)

## Abbreviations

JZSH: Jizushan, a village in Bingchuan county
LXJ and MG respectively: Lixiji and Moguang, two villages in Heqing county
XY: represents Xiangyun county
SY: represents Shiyang, a village in Dayao
JT and QS: represent Jietou and Qushi respectively, two villages in Tengchong county
LWM: little white mushroom, a common local name for *Trogia venenata*.
