# Supplementary figures and images for: Evidence for Inbreeding and Genetic Differentiation among Geographic Populations of the Saprophytic Mushroom Trogia venenata from Southwestern China
Source: PLoS One. 2016 Feb 18;11(2):e0149507. doi: 10.1371/journal.pone.0149507 (PMC4758605; doi:10.1371/journal.pone.0149507)

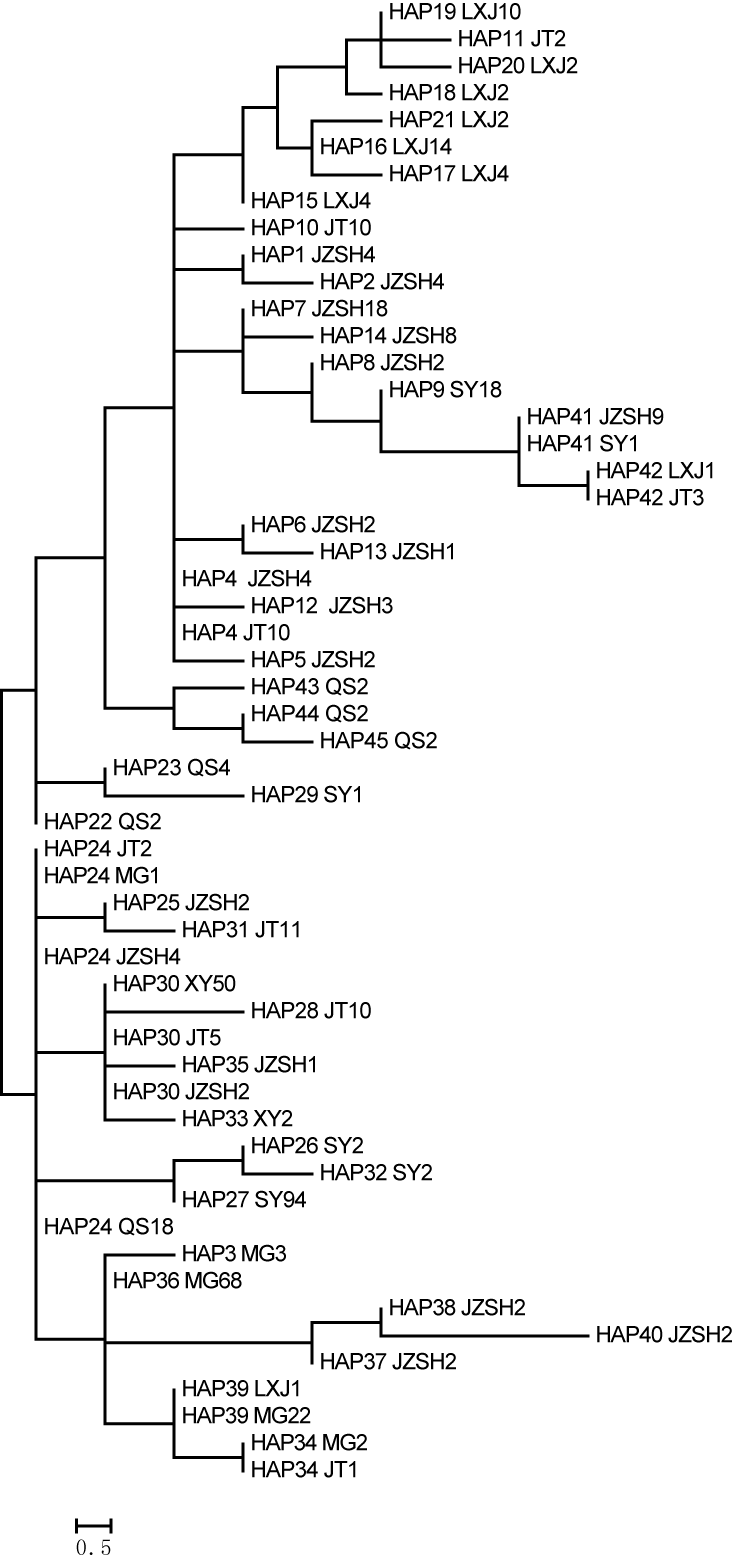

Supplement: S1 Fig — A total of 45 unique rpb2 haplotypes were found in our samples. For each rpb2 haplotype (HAP), the first number represents the haplotype assignment; the characters after represent the county/community from where the strains were sampled; the last number represents the total number of strains belonging to the specific haplotype in that local population. Only representative sequences of unique haplotypes from each location are shown. (TIF) [file pone.0149507.s001.tif]

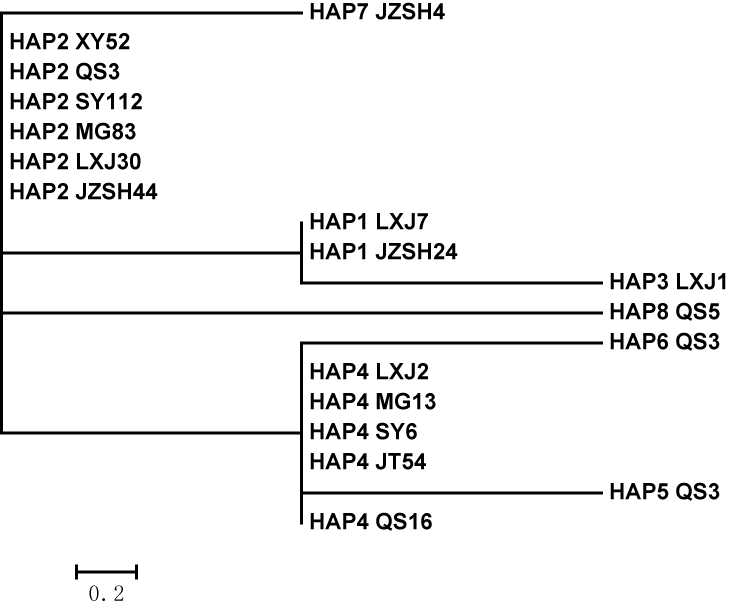

Supplement: S2 Fig — A total of 8 unique tef1-α haplotypes were found in our samples. For each tef1-α haplotype (HAP), the first number represents the haplotype assignment; the characters after represent the county/community from where the strains were sampled; the last number represents the total number of strains belonging to the specific haplotype in that local population. Only representative sequences of unique haplotypes from each location are shown. (TIF) [file pone.0149507.s002.tif]

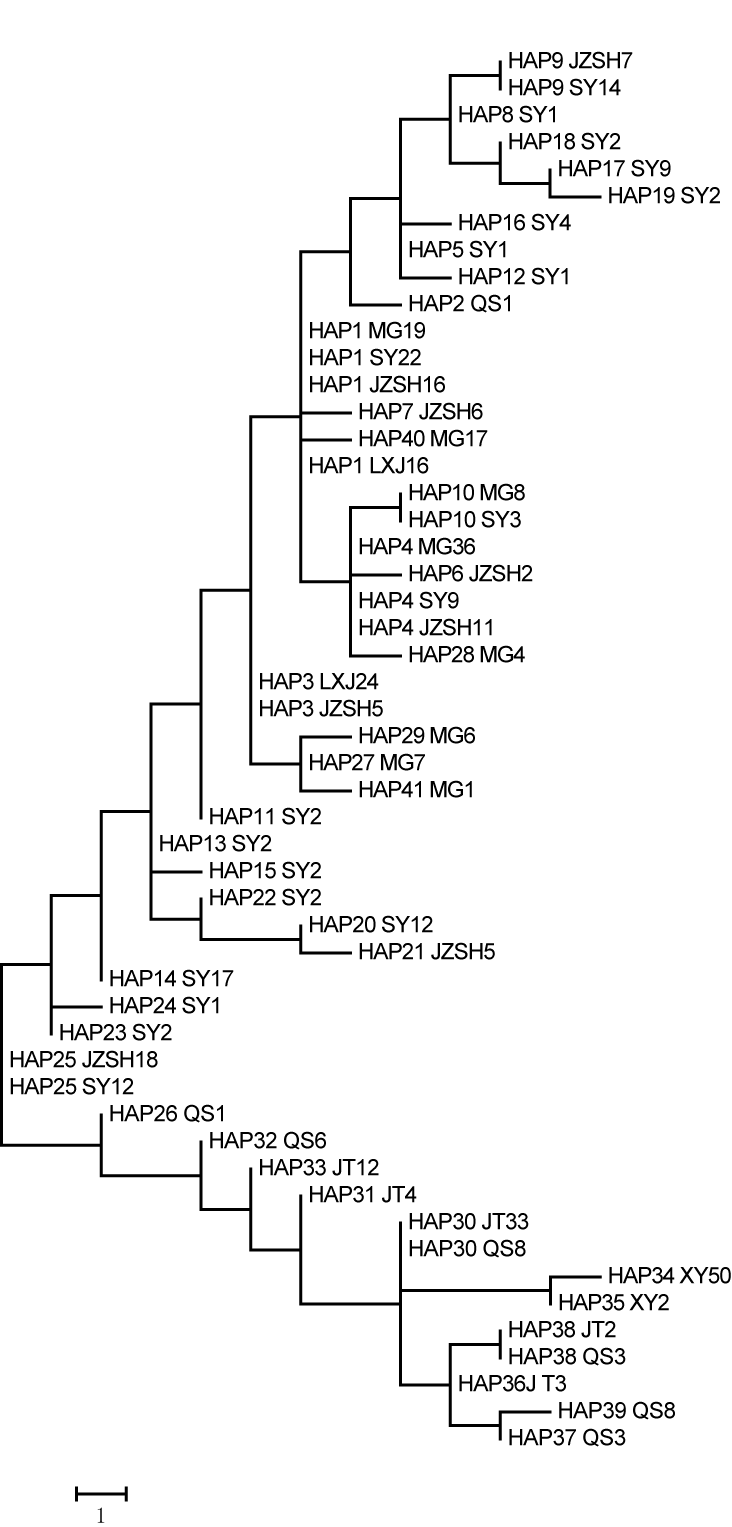

Supplement: S3 Fig — A total of 41 unique β-tub haplotypes were found in our samples. For each β-tub haplotype (HAP), the first number represents the haplotype assignment; the characters after represent the county/community from where the strains were sampled; the last number represents the total number of strains belonging to the specific haplotype in that local population. Only representative sequences of unique haplotypes from each location are shown. (TIF) [file pone.0149507.s003.tif]

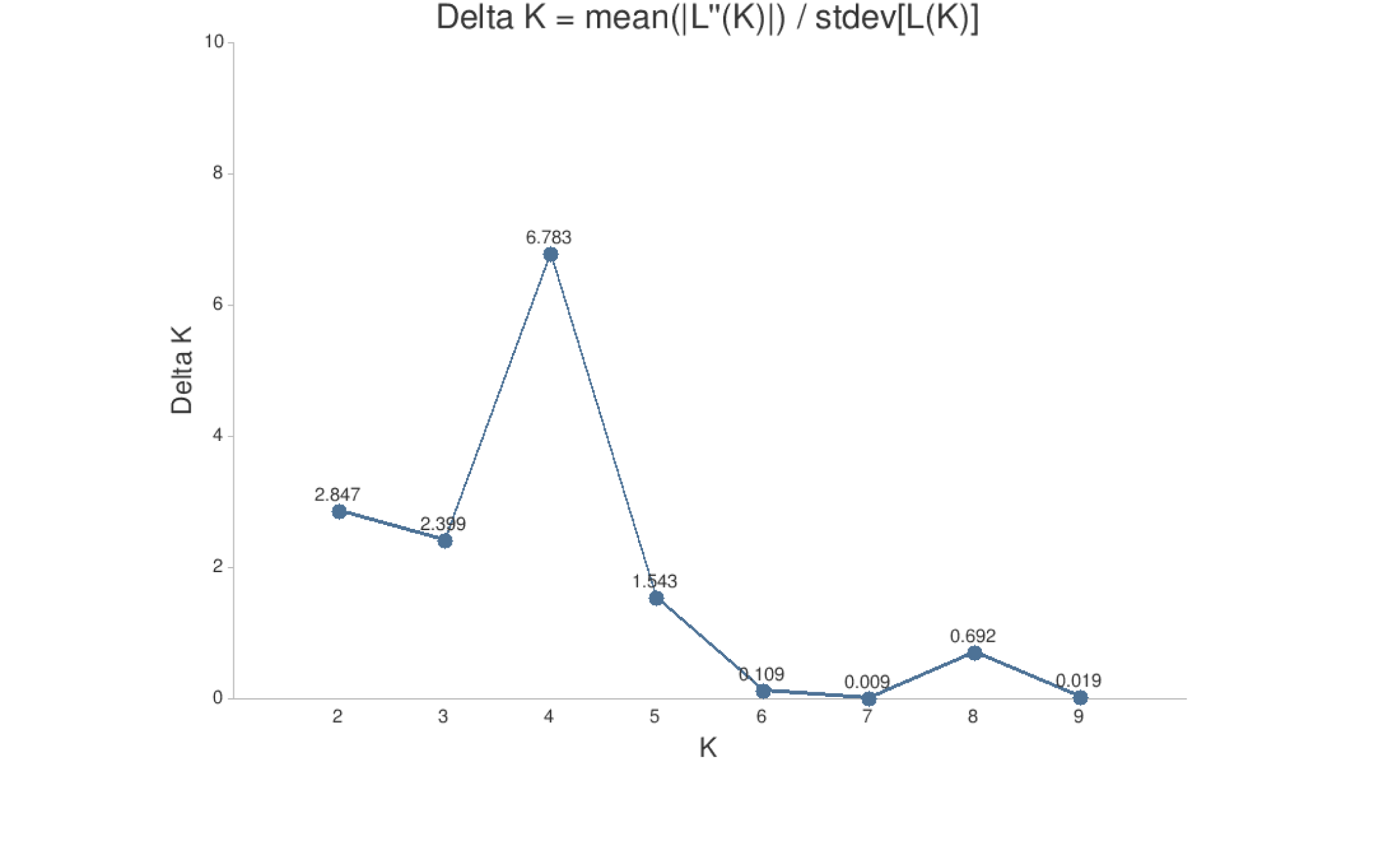

Supplement: S4 Fig — (TIF) [file pone.0149507.s004.tif]
