# Supplementary material for: Evidence for Inbreeding and Genetic Differentiation among Geographic Populations of the Saprophytic Mushroom Trogia venenata from Southwestern China
Source: PLoS One. 2016 Feb 18;11(2):e0149507. doi: 10.1371/journal.pone.0149507 (PMC4758605; doi:10.1371/journal.pone.0149507)
Supplement: S1 Table — (DOCX) [file pone.0149507.s005.docx]

S1 Table. Pairwise *F_ST_* values between geographical populations of *T. venenata* from Yunnan, southwestern China.

| Jizushan | Lixiji | Moguang | Xiangyun | Shiyang | Jietou | Qushi |  |
| --- | --- | --- | --- | --- | --- | --- | --- |
| 0.000 |  |  |  |  |  |  | Jizushan |
| 0.084 | 0.000 |  |  |  |  |  | Lixiji |
| 0.120 | 0.151 | 0.000 |  |  |  |  | Moguang |
| 0.306 | 0.357 | 0.354 | 0.000 |  |  |  | Xiangyun |
| 0.140 | 0.164 | 0.170 | 0.343 | 0.000 |  |  | Shiyang |
| 0.277 | 0.355 | 0.380 | 0.615 | 0.438 | 0.000 |  | Jietou |
| 0.154 | 0.219 | 0.237 | 0.451 | 0.280 | 0.141 | 0.000 | Qushi |
