# Supplementary material for: Evidence for Inbreeding and Genetic Differentiation among Geographic Populations of the Saprophytic Mushroom Trogia venenata from Southwestern China
Source: PLoS One. 2016 Feb 18;11(2):e0149507. doi: 10.1371/journal.pone.0149507 (PMC4758605; doi:10.1371/journal.pone.0149507)
Supplement: S2 Table — (DOCX) [file pone.0149507.s006.docx]

S2 Table. Summary results of AMOVA within and among geographic populations of *Trogia venenata* from Yunnan, southwestern China.

| Three genes combination | df | SS | MS | Estimated  variance | Percentage | Stat | Value | P |
| --- | --- | --- | --- | --- | --- | --- | --- | --- |
| Among Regions | 2 | 204.855 | 102.427 | 0.586 | 14% | PhiRT | 0.136 | 0.001 |
| Among populations within regions | 4 | 184.410 | 46.102 | 1.543 | 36% | PhiPR | 0.415 | 0.001 |
| Within populations | 225 | 490.033 | 2.178 | 2.178 | 51% | PhiPT | 0.494 | 0.001 |
| Total | 231 | 879.297 |  | 4.308 | 100% |  |  |  |

d.f., Degrees of freedom; SS, sum of squared observations; MS, mean of squared observations; Est. var., estimated variance; % Var., percentage of total variance; PhiRT, proportion of the total genetic variance between regions; PhiPR, proportion of the total genetic variance among populations within a region; PhiPT, proportion of the total genetic variance among individuals within populations.
