# Supplementary material for: Evidence for Inbreeding and Genetic Differentiation among Geographic Populations of the Saprophytic Mushroom Trogia venenata from Southwestern China
Source: PLoS One. 2016 Feb 18;11(2):e0149507. doi: 10.1371/journal.pone.0149507 (PMC4758605; doi:10.1371/journal.pone.0149507)
Supplement: S3 Table — (DOCX) [file pone.0149507.s007.docx]

S3 Table. Hardy-Weinberg Equilibrium tests for each geographic population at each locus

| Populations | Locus | DF | ChiSq | P value | Significance |
| --- | --- | --- | --- | --- | --- |
| Jizushan | *rpb2* | 153.000 | 470.111 | 0.000 | *** |
|  | *tef1-α* | 3.000 | 36.298 | 0.000 | *** |
|  | *β-tub* | 36.000 | 143.750 | 0.000 | *** |
| Lixiji | *rpb2* | 36.000 | 160.000 | 0.000 | *** |
|  | *tef1-α* | 6.000 | 20.497 | 0.002 | ** |
|  | *β-tub* | 1.000 | 4.201 | 0.040 | * |
| Moguang | *rpb2* | 10.000 | 98.834 | 0.000 | *** |
|  | *tef1-α* | 1.000 | 0.882 | 0.348 | ns |
|  | *β-tub* | 28.000 | 176.964 | 0.000 | *** |
| Shiyang | *rpb2* | 15.000 | 295.000 | 0.000 | *** |
|  | *tef1-α* | 1.000 | 0.169 | 0.681 | ns |
|  | *β-tub* | 190.000 | 930.492 | 0.000 | *** |
| Jietou | *rpb2* | 36.000 | 162.437 | 0.000 | *** |
|  | *tef1-α* |  |  |  |  |
|  | *β-tub* | 10.000 | 54.620 | 0.000 | *** |
| Qushi | *rpb2* | 15.000 | 75.000 | 0.000 | *** |
|  | *tef1-α* | 10.000 | 39.038 | 0.000 | *** |
|  | *β-tub* | 21.000 | 64.219 | 0.000 | *** |
| Xiangyun | *rpb2* | 1.000 | 26.000 | 0.000 | *** |
|  | *tef1-α* |  |  |  |  |
|  | *β-tub* | 1.000 | 26.000 | 0.000 | *** |

DF, the degrees of freedom; ChiSq, the Chi-squared value; ns＝not significant, * P<0.05, ** P<0.01, *** P<0.001.
