# Supplementary material for: Evidence for Inbreeding and Genetic Differentiation among Geographic Populations of the Saprophytic Mushroom Trogia venenata from Southwestern China
Source: PLoS One. 2016 Feb 18;11(2):e0149507. doi: 10.1371/journal.pone.0149507 (PMC4758605; doi:10.1371/journal.pone.0149507)
Supplement: S4 Table — (DOCX) [file pone.0149507.s008.docx]

S4 Table. GenBank accession numbers for sequences presented in this study

| Gene Name | GenBank Accession Numbers |
| --- | --- |
| Internal transcribed spacer (ITS) regions of the nuclear ribosomal RNA gene cluster | KT967978 to KT968080 |
| The second largest subunit of the nuclear RNA polymerase B gene (rpb2) | KT971373 to KT971604 |
| The translation elongation factor 1-α gene (tef1-α) | KT971605 to KT971836 |
| The β tubulin gene (β-tub) | KT971837 to KT972068 |
